# Pregnancy outcomes in infertile patients undergoing intracytoplasmic sperm injection and two interval programs of hCG-to-oocyte aspiration

**DOI:** 10.25122/jml-2021-0299

**Published:** 2022-12

**Authors:** Milat Ismail Haje, Avin Sadiq Jamil, Nazar Polis Shabila, Danar Darsim Jawad

**Affiliations:** 1 Assisted Reproductive Technology, College of Medicine, Hawler Medical University, Erbil, Iraq; 2 Department of Obstetrics and Gynecology, College of Medicine, Hawler Medical University, Erbil, Iraq; 3 Department of Community Medicine, College of Medicine, Hawler Medical University, Erbil, Iraq

**Keywords:** hCG, intracytoplasmic sperm injection, the pregnancy rate

## Abstract

This study aimed to determine the outcomes of intracytoplasmic sperm injection (ICSI) if oocyte retrieval was done 32–34 hours or 34:05–36 hours after human chorionic gonadotropin (hCG) injection. A randomized sample involving 186 patients with tubal failure was divided into groups A (96 patients) and B (90 patients). Intracytoplasmic sperm injection was performed on all patients according to described protocols. The number of oocytes retrieved, oocyte cumulus complex quality, number of fertilized eggs, and pregnancy rates were compared between groups. The total of oocytes retrieved in group B was significantly higher than in group A but not significant (P=0.068). The oocyte maturation rate was also significantly higher in the long interval group B than in the short interval group A (P=0.039). There was a significant difference between the two groups in terms of fertilization rate (0.040), and the pregnancy rate in group B was higher than that in group A, but it was not significant (P=0.055). The prolonged interval could also increase the pregnancy rate, but it was not significant. It seems that if the interval between hCG priming and oocyte retrieval is prolonged, the percentage of the number of oocytes retrieved, mature oocytes (MII), and fertilized oocytes increases.

## INTRODUCTION

A sequence of crucial procedures occurs during human chorionic gonadotropin (hCG) priming and oocyte retrieval. These processes include the start of luteinization and expansion of cumulus cells. Oocyte resumption meiosis is also accomplished during this interval [1]. Luteinizing hormone (LH), a heterodimeric glycoprotein, includes an alpha chain and a separate beta subunit. It gives biological specificity to the hormone-receptor interaction in target tissues. The anterior pituitary gland produces LH [2]. The natural endogenous LH surge does not usually occur. However, if it occurs, it is done with inappropriate magnitude and timing during controlled ovarian hyperstimulation (COH). As a result, exogenous gonadotropin is required to substitute endogenous LH surge [1].

Gonadotropin is a hormone that affects ovulation timing and menstrual cycle regularities [3]. Human chorionic gonadotropin (hCG) is the commonly applied exogenous LH to stimulate physiologic effects. Also, it has the ability to predict early threatened abortion [4]. According to the World Health Organization (WHO) reports, almost 15% of married women are infertile, and assisted reproductive technology (ART) methods have nearly 25% efficiency [5].

Human chorionic gonadotropin triggers follicular maturation in the ART platform prior to oocyte retrieval [6]. According to WHO, ovulation may happen 24 to 56 hrs after starting the LH surge. The mean time for ovulation is 32 hours [7]. One study demonstrated that natural ovulation after an LH surge might start between 30–36 hours. After hCG injection, the ovulation could occur between 36–40 hours during the superovulation cycle without the GnRH-a use [8]. Taymor et al. [9] revealed that the beginning of ovulation generally starts 36–38 hs after the LH surge introduction in the natural menstrual cycle. Edwards and Steptoe [10] demonstrated in the 1970s that if satisfactory follicular progress emerges, then the start of ovulation can occur 36–38 hours after an hCG injection. The data from Testart and Frydman's study [11] indicate that follicle rupture can happen after 36–38 hours of hCG administration. The pharmacokinetics of hCG and its relation to ovulation was studied by Nader et al. [12], who suggested that ovulation can start before 36 hrs in some females and advised aiming for a <35 hrs interval for ovulation to be evaded. Many *in vitro* fertilization (IVF) plans are usually present for hCG injection. In most IVF programs, the frequently used ovulation interval is 32 to 36 hrs. This interval is obtained from the investigations on patients who applied clomiphene citrate (CC) and/or human menopausal gonadotropin (hMG) for induction of ovulation [8, 11]. CC is a weak synthetic estrogen being used as the primary treatment for infertility and in cases of anovulation. Clomiphene usage leads to ovulation in 80% of patients [13].

The prolonged retrieval of luteinization-to-oocyte can increase oocyte production with a fully expended cumulus, revealing the oocyte's maturation. Subsequently, scientists supposed that the successive percentage of proceeding oocytes towards cleavage and fertilization also improved. This implies that a longer interval might enhance gamete quality through optimal *in vivo* maturation [8]. At the same time, other studies [14, 15] indicated that the difference in IVF treatment cycles' outcomes when the interval is continued is not significant. In a study, the comparison of *in vitro* maturation (IVM) and IVF were clinically investigated for infertile polycystic ovary syndrome (PCOS) patients. Based on the results, IVM had a lower occurrence of mild and severe ovarian hyperstimulation syndrome (OHSS). The implantation and pregnancy rates of IVM were lower than standard IVF [16].

This study aimed to determine if a continued hCG-to-oocyte retrieval interval would have positive intracytoplasmic sperm injection (ICSI) outcomes.

## MATERIAL AND METHODS

The study was conducted at the Fertility and IVF Center of Maternity Teaching Hospital, Erbil, Iraq, between January 2011 and December 2012. Seminal fluid analysis (SFA) (for males) and ultrasound and laparoscopy (for females) were performed to exclude other causes like polycystic ovary, myoma, malformations of the uterus, or endometriosis. Participants were between 20 and 35 years old. Infertile cases with tubal factors were randomly allocated into groups: A (96 patients) and B (90 patients). All other causes of infertility were excluded. The patients underwent intracytoplasmic sperm injection–embryo transfer (ICSI-ET) according to antagonist protocols. Hyperstimulation with hMG (Merional) or follicle-stimulating hormone (FSH) (Gonal f, Serono, Switzerland) was performed. The initial dosage was 225 IU; after 4 days, transvaginal ultrasound U/S and serum estradiol (E2) concentration were used to monitor the ICSI cycle. Accordingly, the antagonist was added to the cycle individually. To induce ovulation, 10,000 IE units hCG (ovitrelle, Serono) were administered subcutaneously once three or more follicles had a diameter exceeding 18 mm. Oocyte aspiration was performed using transvaginal U/S method 32–34 or 34.05–36 hrs after hCG injection was given in groups A and B, respectively. Embryos were checked on days 1, 2, and 3. The transfer was done on day three, and a maximum of four embryos were transferred. All patients received progesterone suppository 800 mg (cyclogest), and serum hCG levels were examined 12 days after transferring the embryo. Also, the rates of implantation and pregnancy in the groups were measured.

### Statistical analysis

The Statistical Package for the Social Sciences (SPSS) version 26 was used for statistical analysis. A P-value below 0.05 was applied to discard the null hypothesis. An independent t-test was applied to compare the mean of the 2 groups.

## RESULTS

The outcomes of the study are summarized in Tables 1–3. There was a difference in the number of oocytes retrieved; however, the difference was not significant. The number of MII (Metaphase II) and fertilized eggs significantly increased with the prolongation of retrieval time. In addition, there were more matured eggs when the time of hCG administration was prolonged to 36 hours. Additionally, a difference was observed between pregnancy rates that increased with the prolongation of time between hCG administration and oocyte retrieval.

**Table 1:** Time of oocytes retrieval among groups.

Time of retrieval	Frequency	Percent
**After 32–34h**	96	51
**After 34–36h**	90	49
**Total**	186	100.0

**Table 2:** Results of ICSI concerning oocyte retrieved 32–34 hours and 34.05 to 36 hs after administering hCG.

Outcome	Time of retrieval				P-value
	After 32h–34h (Group A)		After 34.05h–36h (Group B)		
	No.	SD	No.	SD	
**Number of eggs**	10.99	5.42	13.05	6.95	0.068
**Number of MII**	8.84	4.43	10.77	5.81	0.039
**Number of fertilized eggs**	7.25	3.72	8.82	4.60	0.040

**Table 3:** Pregnancy rate 32–34 hours and 34.05 to 36 hours after hCG administration.

Time of retrieval	Pregnancy rate				P-value
	Negative		Positive		
	No.	%	No.	%	
**After 32–34h (Group A)**	55	57.9%	40	42.1%	0.055
**After 34.05–36h (Group B)**	34	39.5%	66	60.5%	

## DISCUSSION

The maturation of oocytes is the key to successful fertilization and proper embryo development. The optimum time interval between hCG administration and the maturation of oocytes is convenient. Countless studies showed different results for all elements studied in this paper: number of eggs, MII, fertilized eggs, and pregnancy rates. However, most studies concluded that prolonging the time interval of oocyte retrieval does not significantly affect the pregnancy rate and the number of eggs retrieved, as is also evident in this study. On the other hand, the quantity of MII and fertilized eggs indicated a significant change when the time interval after hCG injection was prolonged to 36 hours. Additionally, drawing a large sample could demonstrate whether the difference in pregnancy rates extending the interval could be significant.

In the present research, all patients had tubal failure, which is an indication of ICSI. This factor should be considered when interpreting this study's results as patients with other symptoms or conditions, such as the male factor, were omitted. The ages of the participants must also be considered, as the quality of oocytes degrades as women get older. The rate of metabolism in the cytoplasm decreases, DNA mutation increases, adenosine triphosphate (ATP) decreases, active oxygen decreases, and aneuploidy increases; hence, the developmental potential of oocytes and embryos decreases [17].

However, elective single embryo transfer is only possible if a top-quality embryo exists. Such embryo transfer provides an acceptable pregnancy rate. The pregnancy rate for one transfer is 40%, and the cumulative pregnancy rate is 60%, higher than double-embryo transmission [17–19]. Top-quality embryos have characteristics such as cleavage velocity and morphological distribution, the lack of multinucleated blastomeres, four or five blastomeres on day 2, seven or more cells on day 3, and ≤20% anucleated fragments [20]. On day two, 42 hours after insemination, most embryos chosen for transfer were in the fourth cycle stage. It is clear that the fourth cell embryo on day two of its cycle is more likely to be good on day three, but 3-cell and greater than 4-cell embryos have low development potential [9].

In human oocytes, a phase of intense nuclear and cytoplasmic activity occurs in the hours following a luteinizing stimulation. As a result, the degree of cellular and cytogenic maturation is most likely dependent on the time between hCG priming and oocyte retrieval. A longer gap would have a bigger intrafollicular effect, create oocytes with complete cellular development, provide more *in vivo* maturation, and have a higher likelihood of developing into 2PN zygotes and high-quality normal cleaving embryos [21–23]. Because immature oocytes firmly adhere to the follicular wall, they are not aspirated easily. This attachment becomes weak, and oocyte retrieval becomes easy when they become mature [14]. One of the significant factors for successful fertilization is oocyte maturation. Delaying oocyte retrieval to a longer interval will allow more oocyte maturation but without any significant effect on the number of eggs and pregnancy rate [1].

A level of E2 that is too much increased is not good for embryo nidification during the endocrine profile. During controlled ovarian hyperstimulation (COH), raised LH may yield granulosa cells luteinized prematurely, affecting the embryo quality and outcomes of pregnancy [11]. Immunity elements like the existence of different antibodies, including anti-sperm, endometrial, ovarian, cardiolipin, and human chorionic gonadotropin antibodies, binding to corresponding antigens can lead to antigenic reactions, trigger the complement system, cause autoimmune reactions, and eventually affect pregnancy events [14, 24]. So, we have to consider these variables adequately when performing ARTs. Oocyte retrieval could be done at any moment following hCG priming within a relatively broad timeframe. This adaptability in hCG-to-oocyte retrieval makes it easier for both patients and clinicians. The findings also suggest that it is critical to understand the temporal relationship between LH surge, follicle rupture, oocyte recovery, and the likelihood of a successful pregnancy during natural and artificially aided menstrual cycles.

## CONCLUSION

The results show that extending the time between hCG priming and oocyte retrieval may increase the number of fertilized eggs and MII. The number of oocytes and pregnancy rates are not significantly different between oocytes retrieved 32–34 hours after hCG injection and oocytes recovered 34:05–36 hours after hCG injection. Even if evidence emerges to confirm the findings, the conclusion should be based on the results of randomized controlled trials with large samples from different races, countries, and regions.

## Personal thanks

We wish to thank all staff of the IVF Center of Maternity Teaching Hospital.
